# Glycocalyx and Endothelial Biomarkers as Prognostic Indicators in Sepsis: A Systematic Review and Meta‐Analysis

**DOI:** 10.1002/mbo3.70155

**Published:** 2025-11-06

**Authors:** Kamila R. Daniyarova, Zhanslu N. Sarkulova, Amin Tamadon, Ainur B. Tokshilykova, Marat N. Sarkulov, Botagoz M. Kalieva, Nadiar M. Mussin, Ramazon Safarzoda Sharoffidin

**Affiliations:** ^1^ Department of Anesthesiology and Reanimatology West Kazakhstan Marat Ospanov Medical University Aktobe Kazakhstan; ^2^ Department of Natural Sciences West Kazakhstan Marat Ospanov Medical University Aktobe Kazakhstan; ^3^ Department of Surgical Diseases No. 2 with a course in Urology West Kazakhstan Marat Ospanov Medical University Aktobe Kazakhstan; ^4^ Department of Surgery No. 2 West Kazakhstan Marat Ospanov Medical University Aktobe Kazakhstan; ^5^ Department of Pharmaceutical Technology Avicenna Tajik State Medical University Dushanbe Tajikistan

**Keywords:** endocan, endothelial dysfunction, glycocalyx, meta‐analysis, mortality, sepsis, Syndecan‐1

## Abstract

Sepsis‐induced endothelial dysfunction, marked by degradation of the endothelial glycocalyx and activation of endothelial cells, plays a pivotal role in the progression to organ failure and mortality. Biomarkers reflecting glycocalyx damage have demonstrated prognostic potential; however, their associations with clinical outcomes remain variable. We systematically evaluated the prognostic utility of glycocalyx‐associated biomarkers (syndecan‐1, heparan sulfate, hyaluronate) and the endothelial activation marker endocan in sepsis with respect to mortality, organ dysfunction, and inter‐study heterogeneity. We included 23 studies through May 2025 encompassing 4529 patients with sepsis. Two independent reviewers extracted data using standardized protocols, including biomarker concentrations and clinical outcomes such as mortality, multiple organ dysfunction syndrome, and respiratory failure. Risk of bias was assessed using the NOS, with 75% of studies rated as low risk. Elevated syndecan‐1 was significantly associated with increased mortality (nine studies, *n* = 2167; OR 2.04, 95% CI, 1.66–2.51; *p* < 0.05; *I*² = 84%). Similarly, elevated endocan predicted mortality with a stronger effect size (six studies, *n* = 435; OR 5.06, 95% CI, 2.52–10.18; *p* < 0.05) and low heterogeneity. In contrast, syndecan‐1 levels were not significantly associated with multiple organ dysfunction syndrome (OR 2.35, 95% CI, 0.93–5.94; *I*² = 94%) or respiratory failure (OR 1.05, 95% CI, 0.27–4.02; *I*² = 91%). The majority of studies were ICU‐based (78.3%), primarily adult cohorts (91.3%), with syndecan‐1 the most commonly assessed biomarker (65.2%). Syndecan‐1 and endocan serve as prognostic biomarkers for mortality in sepsis, with endocan demonstrating greater inter‐study consistency.

## Graphical abstract

Elevated circulating levels of glycocalyx‐associated biomarkers—syndecan‐1 and endocan—predict higher mortality in sepsis. This meta‐analysis identifies endothelial injury as a key prognostic mechanism linking glycocalyx degradation to multiorgan dysfunction, supporting the use of endothelial biomarkers for early risk stratification and precision therapy in critically ill patients.

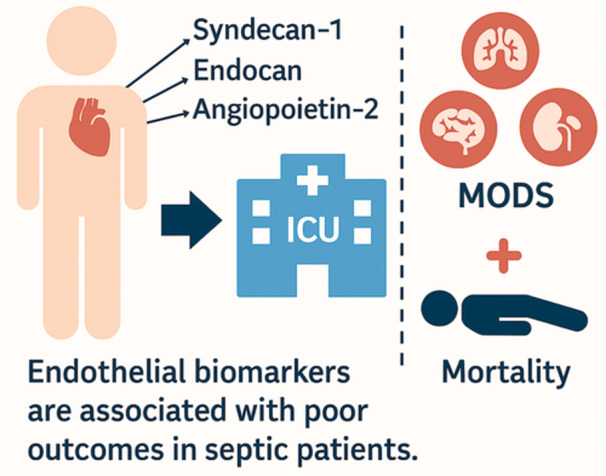

## 1 Introduction

Sepsis remains a major global health challenge, responsible for an estimated 48.9 million cases and 11 million deaths annually (Rudd et al. 2020). A critical component of sepsis pathophysiology is endothelial dysfunction, driven in part by degradation of the endothelial glycocalyx—a protective layer of proteoglycans and glycosaminoglycans that regulates vascular permeability, leukocyte adhesion, and coagulation (Lipowsky 2012; Reitsma et al. 2007). Inflammatory mediators, enzymatic activity, and oxidative stress in sepsis lead to the shedding of glycocalyx constituents such as syndecan‐1, heparan sulfate, and hyaluronate (Schmidt et al. 2012). Concurrently, endothelial activation is marked by elevated levels of endocan, a proteoglycan secreted in response to vascular inflammation (Pauly et al. 2016).

Emerging evidence links elevated syndecan‐1 and endocan levels to increased mortality in sepsis (Ikeda et al. 2018; Johansen et al. 2015). However, association with organ dysfunction—such as multiple organ dysfunction syndrome and respiratory failure—remain inconsistent (Ostrowski et al. 2015). While previous meta‐analyses have examined individual biomarkers, none have comprehensively synthesized the prognostic roles of both glycocalyx degradation and endothelial activation markers in relation to sepsis outcomes. Moreover, recent studies have introduced glycocalyx‐targeted therapies such as heparanase inhibitors and plasma‐based resuscitation (Chen et al. 2024; Clausen et al. 2024), underscoring the need for an updated analysis.

This systematic review and meta‐analysis aimed to: A) Evaluate the association between glycocalyx damage markers (syndecan‐1, heparan sulfate, hyaluronate) and endothelial activation (endocan) with mortality in sepsis. B) Assess whether these biomarkers predict multiple organ dysfunction syndrome, respiratory failure, or acute kidney injury. C) Explore heterogeneity across studies and identify potential sources of variation in biomarker performance.

## 2 Materials and Methods

### 2.1 Search Strategy and Study Selection

A comprehensive literature search was conducted across major medical databases without language restrictions. This systematic review was prospectively registered on PROSPERO (registration number: CRD420251060872). The review was conducted and reported in accordance with the PRISMA and MOOSE guidelines.

Studies were eligible for inclusion if they involved pediatric patients ( ≥ 1 month old) or adults with sepsis of any etiology or severity, including sepsis‐associated organ dysfunction and septic shock. The definitions of sepsis were based on either the Surviving Sepsis Campaign (ACCP/SCCM Consensus Conference) or the Sepsis 3 criteria for adults.

### 2.2 Inclusion and Exclusion Criteria

Included studies measured serum biomarkers indicative of glycocalyx injury—such as syndecan‐1, heparan sulfate, and hyaluronate—and endothelial activation, specifically endocan, within 48 h of hospital admission (emergency department, general ward, or intensive care unit). Only human studies reporting sepsis‐related clinical outcomes, including mortality, ICU or hospital length of stay, organ failure, and vasopressor use, were considered. Studies employing video microscopy were excluded because this technique evaluates structural glycocalyx thickness rather than circulating biomarkers, and the lack of standardized quantitative thresholds could confound biomarker‐based meta‐analysis.

Both peer‐reviewed and gray literature were reviewed regardless of study design. Exclusion criteria included preclinical studies, studies involving pregnant women, letters to the editor, narrative reviews, and studies that did not report clinical outcome data.

### 2.3 Data Sources and Search Terms

The literature search was conducted using the Scopus and Web of Science databases up to May 25, 2025. Key MeSH terms are shown in Table 1. In cases where full‐texts or essential data were not readily available, the authors were contacted to obtain additional information.

**Table 1:** Search strategy and query terms used for identifying studies on glycocalyx damage and endothelial activation in sepsis.

Code	Query
#1	“sepsis” OR “septic shock”
#2	“endothelium” OR “syndecan‐1” OR “syndecans” OR “CD138” OR “CD138 antigens” OR “heparan sulfate” OR “syndecan 4” OR “ryudocan” OR “endocan” OR “endothelial specific molecule 1” OR “ESM‐1 protein” OR “endocan protein” OR “glycocalyx”
#3	“epidemiological studies” OR “clinical trial”
#4	“mortality” OR “Death” OR “length of stay” OR “hospital stay” OR “vasopressor” OR “vasoactive score”
#5	#1 AND #2 AND #3 AND #4

### 2.4 Study Selection and Data Processing

Initially, titles and abstracts were screened, followed by a full‐text review of studies meeting the inclusion criteria (K.R.D., A.T.). Data were extracted from eligible studies, with any uncertainties regarding eligibility resolved by a second investigator (A.T.). A standardized extraction form was developed by one author (K.R.D.) and applied to the selected studies. The remaining authors (N.M.M., Zh.N.S., A.B.T., B.M.K., M.N.S., R.S.S.) verified both the extraction form and the extracted data. The collected data, presented in Table 2, included biomarkers measured in ng/mL using the ELISA technique.

**Table 2:** Studies assessing glycocalyx damage in sepsis patients.

Author, year, citation	Country	Age (IQR/median)	Sample size	Biomarkers assessed	Assessment timing	Clinical condition	Primary outcome	Secondary outcomes	Risk of bias
(Beurskens et al. 2020)	Netherlands	67 (61–76)	21	Syndecan‐1	Upon ICU admission	Septic shock	Mortality	MODS	*******
(Chen et al. 2023)	China	61 (45–72)	95	Syndecan‐1	Days 1, 3, and 7 posttreatment	Sepsis with acute GI injury	Improvement in AGI grade	Coagulation and immune markers; 28‐day survival	*******
(Chen et al. 2024)	China	63 (55.5–70.5)	105	Syndecan‐1	Days 1, 3, and 7 posttreatment	Septic cardiomyopathy	Cardiac function (LVEF, FS)	Inflammatory markers; 28‐day mortality	*******
(Clausen et al. 2024)	Denmark	69 (NR)	44	Syndecan‐1, VEGFR1, sTM, sE‐selectin, Histone complexes	Baseline and 24 h post‐resuscitation	Severe septic shock (SOFA 13)	Biomarker and microvascular changes (PPV/PVD)	90‐day mortality; SOFA; CRRT‐free and vasopressor‐free days	***
(Holzmann et al. 2018)	Germany	61 (51–70)	55	Syndecan‐1	Days 1 and 2	Postoperative sepsis	Mortality	MODS; ICU stay	*******
(Ikeda et al. 2018)	Japan	73 (65–81)	54	Syndecan‐1	At admission and days 1, 2, 4	Septic shock	Mortality	Coagulopathy; RF; MODS	********
(Inkinen et al. 2019)	Finland	66 (65–75)	619	Syndecan‐1	24 h after ICU admission	Sepsis	Mortality	AKI; fluid overload	*********
(Johansen et al. 2015)	Denmark	65 (58–75)	1113	Syndecan‐1	Upon admission	Septic shock	Mortality	MODS	*******
(Ioakeimidou et al. 2017)	Greece	69 (48–90)	175	Endocan	ICU admission and 24 h later	Sepsis	Temporal biomarker dynamics	MODS	*****
(Mihajlovic et al. 2014)	Serbia	61 (43–79)	60	Endocan	24 h post‐onset	Sepsis	MOFS, mortality	Coagulation tests (PT, PTT)	*******
(Nelson et al. 2008)	Sweden	65 (28–87)	36	Syndecan‐1	ICU admission	Septic shock	Mortality	MODS	***
(Nelson et al. 2017)	Sweden	67 (53–75)	137	Syndecans 1–4	PICU admission	Critical illness	Mortality	MODS	********
(Orbegozo et al. 2017)	Belgium	61 (44–78)	96	Endocan	ARDS diagnosis and following morning	ARDS	Mortality	Ventilation duration; hospital stay	*******
(Ostrowski et al. 2015)	Denmark	61 (46–73)	321	Syndecan‐1	Before antibiotic therapy	Severe sepsis	Mortality	MODS; ICU stay	*******
(Palud et al. 2015)	France	61 (54–68)	20	Endocan	ICU admission	Septic shock	MOFS	NR	*****
(Pauly et al. 2016)	Germany	57 (26–88)	150	Endocan	Days 1, 3, 8 post‐ICU	Severe sepsis/septic shock	30‐day mortality	6‐month mortality	*******
(Piotti et al. 2021)	Italy	68 (58–77)	375	Syndecan‐1, S1P, VE‐cadherin	Days 1, 2, and 7 after ICU admission	Septic shock (ALBIOS)	Biomarker relation to organ failure and 90‐day mortality	RRT; coagulation failure; SOFA subscores	****
(Qian et al. 2025)	China	62 (30–93)	74	Syndecan‐1	Days 0, 1, and 2 post‐ICU	Sepsis (Sepsis‐3)	30‐day mortality	90‐day mortality; AKI; ARDS; CRRT	***
(Smart et al. 2017)	Australia	59 (52–66)	138	Syndecan‐1, Hyaluronate	Admission, 3 h, 24 h	Sepsis/infection	Mortality	MODS; ICU referral	********
(Smart et al. 2018)	Australia	66 (61–71)	72	Syndecan‐1, Endocan, Hyaluronate	ER admission and multiple timepoints	Pneumonia with septic shock	Mortality	RF; ventilation need	*******
(Wang et al. 2022)	China	60 (42–79)	40	Syndecan‐1, Ang‐2, ET‐1	Baseline and at 24, 72, 120 h	Septic shock	Microcirculatory indices (TVD, PVD, MFI)	28‐day mortality; SOFA; vasopressor use; ICU stay	*******
(Wei et al. 2018)	USA	40 (30–51)	512	Syndecan‐1	4 h post‐admission	Trauma with sepsis	New‐onset sepsis	MODS; hospital stay	********
(Whitney et al. 2020)	USA	5 (4–13)	119	Endocan	72 h, day 6, day 14	Pediatric sepsis ± ARDS	Multiorgan dysfunction, mortality	NR	******

Abbreviations: AKI, acute kidney injury; ARDS, acute respiratory distress syndrome; CRRT, continuous renal replacement therapy; ER, Emergency room; ICU, intensive care unit; IQR, interquartile range; MODS, multiple organ dysfunction syndrome; NR, not reported; PICU, pediatric ICU; PT, prothrombin time; PTT, partial thromboplastin time.

*Evaluated using the Newcastle‐Ottawa scale.

### 2.5 Methodological Quality Assessment

Study quality was evaluated based on external validity (target population, generalizability, and random error) and internal validity.

### 2.6 Outcomes

The primary outcome assessed all‐cause mortality risk at any point during hospitalization in sepsis and septic shock patients with altered glycocalyx injury and endothelial activation biomarkers. Sepsis was defined as a systemic viral or bacterial infection leading to organ dysfunction, while septic shock included sepsis with cardiovascular dysfunction causing hypotension, perfusion deficits, or requiring vasoactive drugs. These criteria applied to both pediatric and adult patients. Studies used either the Sepsis‐2 (1992 ACCP/SCCM) or Sepsis‐3 (2016) definitions; differences were recorded and considered in sensitivity analyses.

Secondary outcomes included the risk of multiple organ dysfunction syndrome, respiratory failure, or acute kidney injury in patients with altered glycocalyx and endothelial biomarkers. Multiple organ dysfunction syndrome was defined as dysfunction in two or more organs (SOFA score > 2), and respiratory failure was identified by a need for > 50% inspired oxygen or mechanical ventilation during hospitalization.

### 2.7 Data Analysis

Heterogeneity sources were examined based on study design, sampling methods, and exposure measurement. The Higgins index (*I*² > 20%) determined heterogeneity. Medians, ranges, and interquartile ranges (IQRs) were converted to means and standard deviations using Wan et al.'s method. Results were reported as odds ratios (ORs) with 95% confidence intervals (95% CI) for categorical variables and mean differences (95% CI) for continuous variables. Non‐normally distributed continuous variables were analyzed using pooled medians and compared via the Mann‐Whitney U test. The Mantel‐Haenszel random‐effects model was applied, with fixed‐effects for low‐heterogeneity studies. Statistical significance was set at *p* < 0.05, and analyses were conducted using RStudio packages.

## 3 Results

### 3.1 Study Selection Process

**Figure 1:**
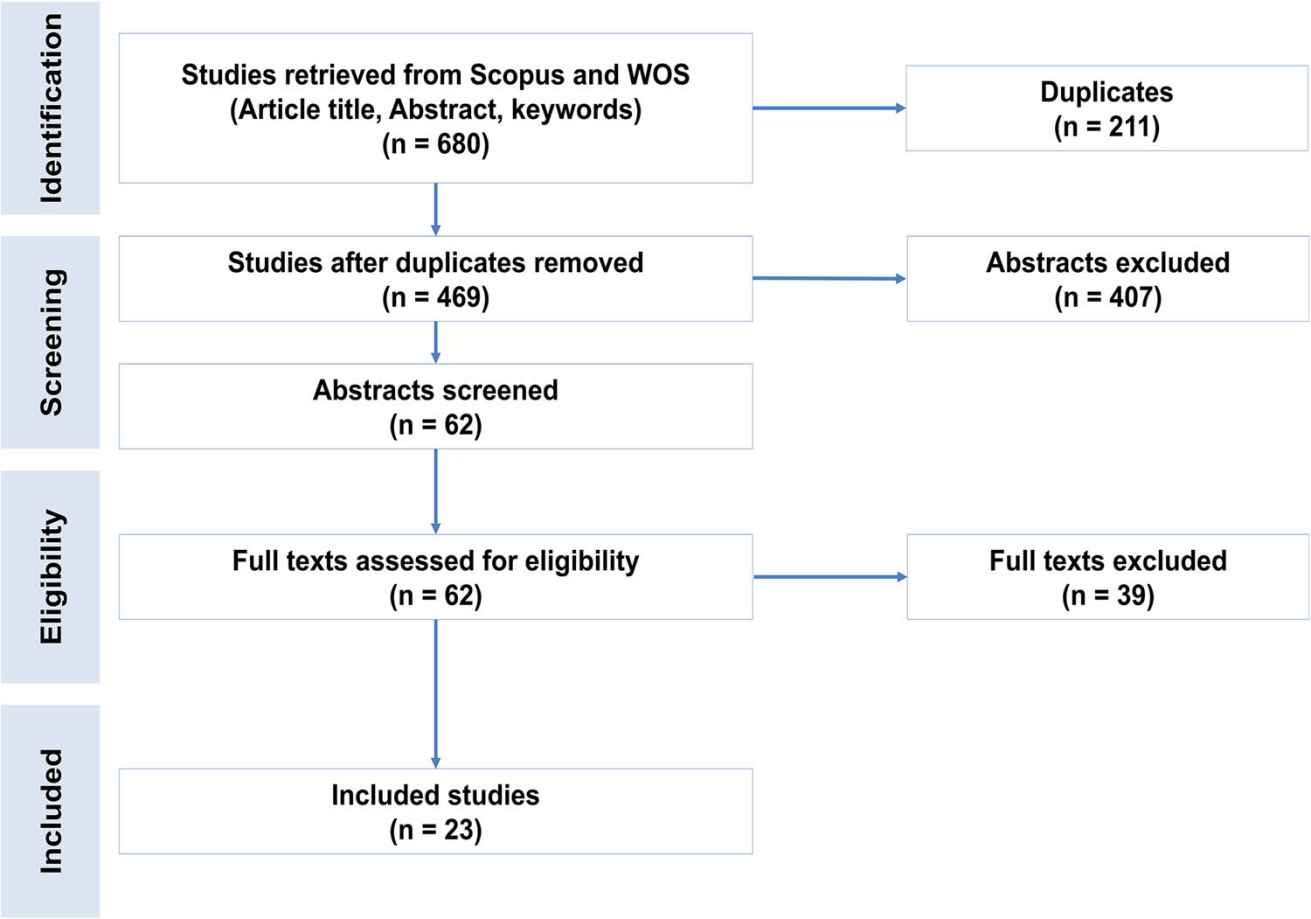
Flowchart of study selection based on PRISMA guidelines for systematic reviews and meta‐analyses.

The initial database search identified 680 studies from Scopus and Web of Science (Figure 1). After removing 211 duplicates, 469 records underwent abstract screening, yielding 62 full‐text articles for eligibility assessment. Following exclusions, 23 studies met inclusion criteria for qualitative synthesis, with 23 studies ultimately included in the meta‐analysis after further refinement.

### 3.2 Risk of Bias Assessment

**Figure 2:**
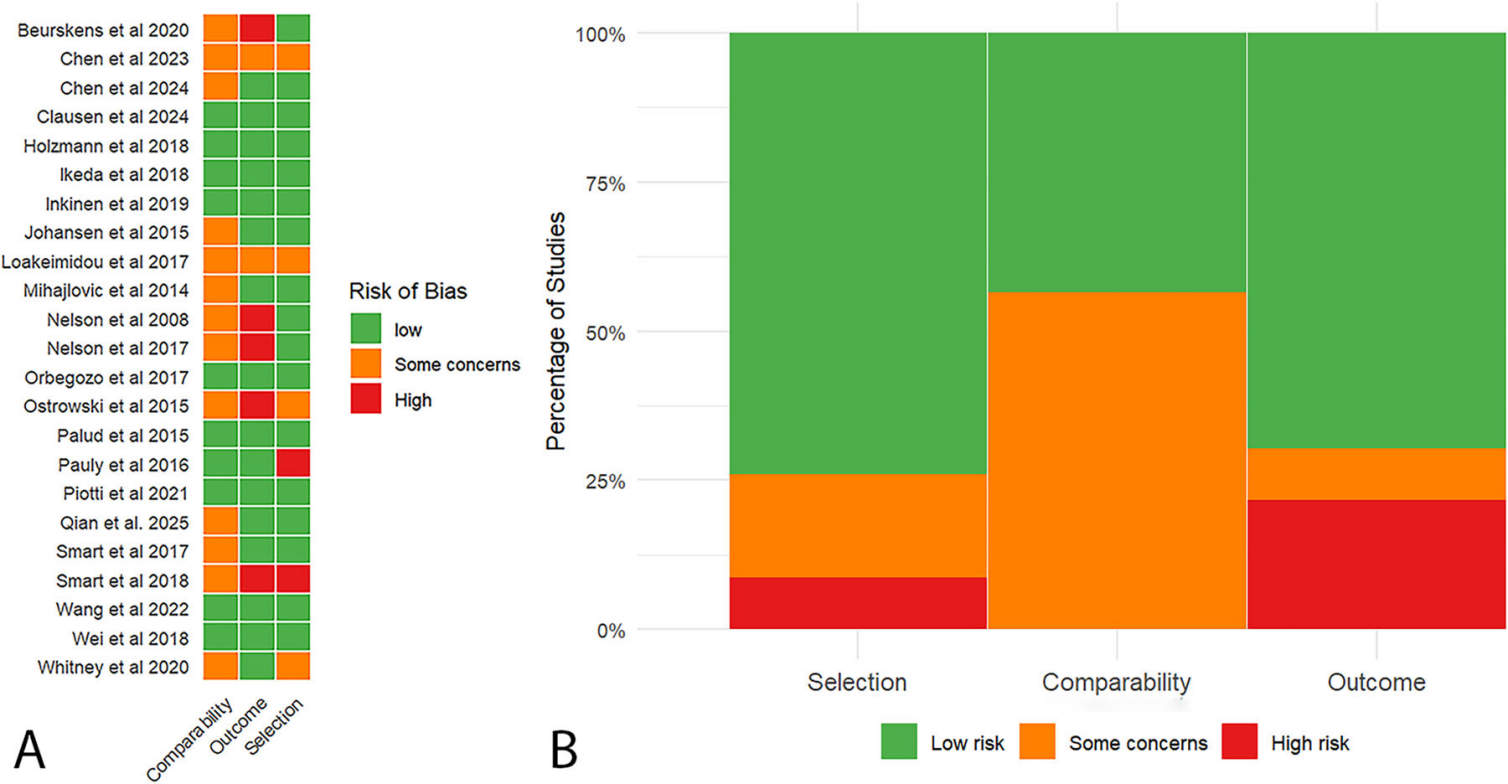
Evaluation of bias risk in included studies: (a) Newcastle Ottawa scale assessment and (b) summary of bias risk.

The methodological quality of the included studies was assessed using the Newcastle‐Ottawa Scale (NOS) for cohort studies, with the results summarized in Figure 2. The evaluation focused on three domains: selection (representativeness of cohorts, ascertainment of exposure, and confirmation that the outcome was not present at baseline), comparability (control for confounding factors), and outcome (assessment of outcomes and adequacy of follow‐up).

Overall, 75% of the studies were judged to have a low risk of bias across all domains, indicating robust methodology in participant selection, exposure determination, and outcome measurement. In contrast, 25% of studies raised some concerns, primarily within the comparability domain due to incomplete adjustment for key confounders such as age, sex, or comorbid conditions. Importantly, none of the included studies were rated as having a high risk of bias in any domain. Domain‐specific analysis showed that 85% of studies demonstrated low risk in the selection domain, reflecting appropriate cohort definitions and reliable exposure ascertainment. In the comparability domain, 65% of studies adequately controlled for important confounding variables, while the remaining studies had limited or unclear confounder adjustment. For the outcome domain, 90% of studies used validated outcome measures and appropriate follow‐up durations. These findings suggest that while the overall methodological quality of the included studies was high, the presence of some limitations in confounder control should be considered when interpreting the results. Nevertheless, the generally low risk of bias across studies supports the reliability and validity of the pooled estimates in this meta‐analysis.

### 3.3 Study Characteristics

This systematic review included 23 studies that investigated glycocalyx injury biomarkers in patients with sepsis, with key details summarized in Table 2. The studies were conducted across 10 countries between 2008 and 2025, involving a total of 4,529 participants, with individual study sample sizes ranging from 20 to 1,113 patients (median: 95 per study). The majority of studies (21 studies, 91.3%) focused on adult populations, where the median age ranged from 57 to 73 years. Two studies (8.7%) included pediatric populations, with median ages between 5 and 13 years.

In terms of biomarker assessment, Syndecan‐1 was the most frequently studied marker, evaluated in 15 studies (65.2%). Endocan was assessed in 7 studies (30.4%), while other glycocalyx‐related biomarkers—such as hyaluronate, syndecans‐2/3/4, and heparan sulfate—were reported in 4 studies (17.4%). Most studies (18 studies, 78.3%) were conducted in intensive care unit settings, while 3 studies (13.0%) recruited patients from emergency departments, and 2 studies (8.7%) involved mixed hospital populations.

The primary clinical outcomes included mortality (28‐, 30‐, or 90‐day), which was assessed in 18 studies (78.3%), and multiple organ dysfunction syndrome, reported in 12 studies (52.2%). Secondary outcomes included respiratory failure, examined in 8 studies (34.8%), and acute kidney injury, assessed in 5 studies (21.7%).

All included studies used observational designs, comprising 19 cohort studies and 4 case‐control studies. The timing of biomarker measurement varied across studies, ranging from emergency department admission to 7 days post‐ICU admission. Six studies (26.1%) employed multicenter designs, and 7 studies (30.4%) included control groups for comparison.

As previously described, the risk of bias assessment using the NOS revealed that 75% of the studies had a low risk of bias across key domains, while 25% showed some concerns, particularly in terms of comparability due to insufficient adjustment for confounding factors. No studies were rated as having a high risk of bias.

### 3.4 Syndecan‐1 and Mortality in Sepsis

**Figure 3:**
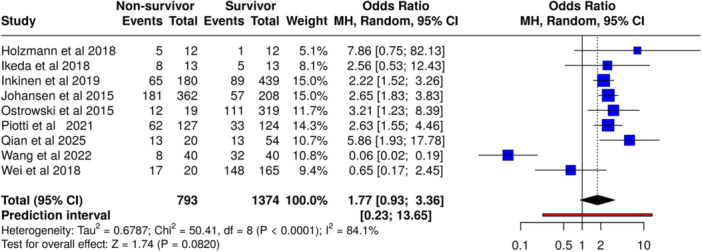
Association between elevated syndecan‐1 levels and mortality in sepsis. Random‐effects model (Mantel–Haenszel); OR = 1.77 [0.93–3.36]; I² = 84.1%.

A total of nine studies (*n* = 2,167 patients, including 793 non‐survivors and 1374 survivors) were analyzed (Figure 3). Using a fixed‐effect model (Mantel‐Haenszel method), elevated syndecan‐1 levels were not significantly associated with increased mortality risk in sepsis patients (OR 2.04, 95% CI, 1.66–2.51, *p* = 0.08). However, substantial heterogeneity was observed (*I*² = 84%, *p* < 0.01), indicating variability in effect sizes across studies beyond chance.

### 3.5 Syndecan‐1 and Multiple Organ Dysfunction Syndrome

**Figure 4:**
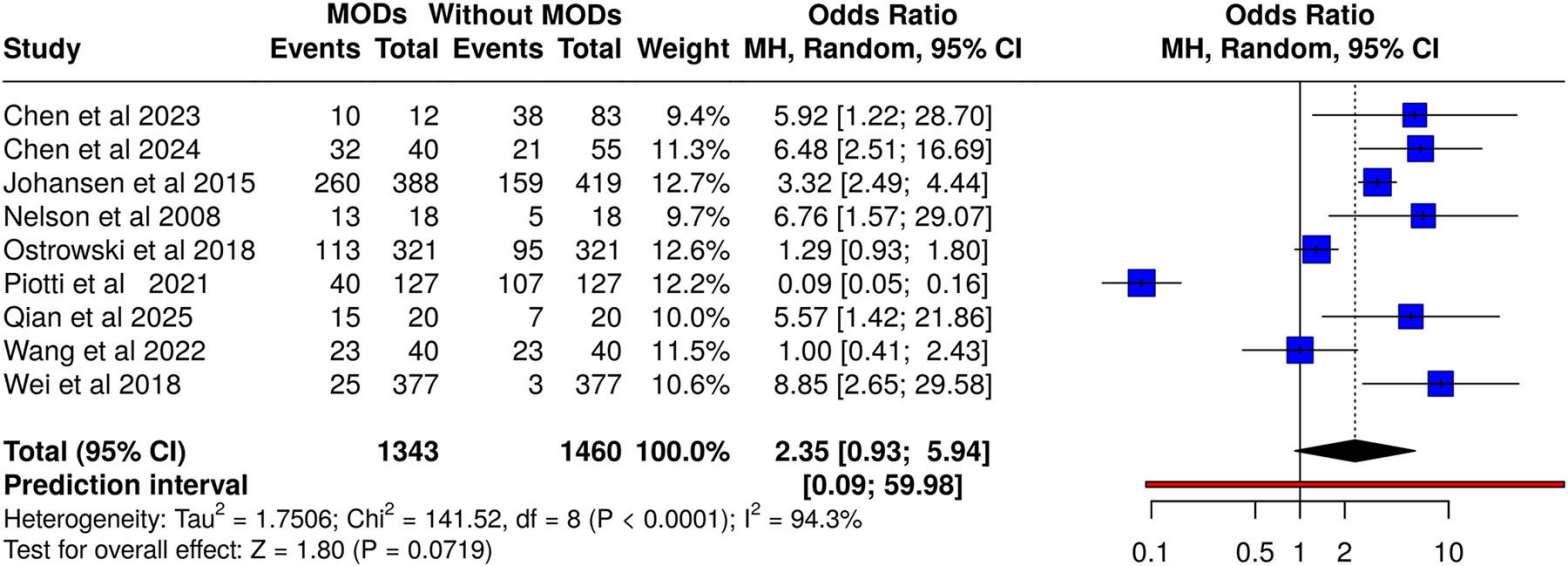
Association between elevated syndecan‐1 levels and multiple‐organ‐dysfunction‐syndrome (MODS) in sepsis. Random‐effects model; OR = 2.35 [0.93–5.94]; *I*² = 94.3%.

Analysis of nine studies (*n* = 2,803 patients, including 1343 with multiple organ dysfunction syndrome and 1460 without multiple organ dysfunction syndrome) found no significant association between syndecan‐1 and multiple organ dysfunction syndrome (OR 2.35, 95% CI, 0.93–5.94, *p* = NS) under a random‐effects model (Figure 4). High heterogeneity was present (*I*² = 94%, *p* < 0.01), suggesting inconsistent effects across studies.

### 3.6 Syndecan‐1 and Respiratory Failure

**Figure 5:**
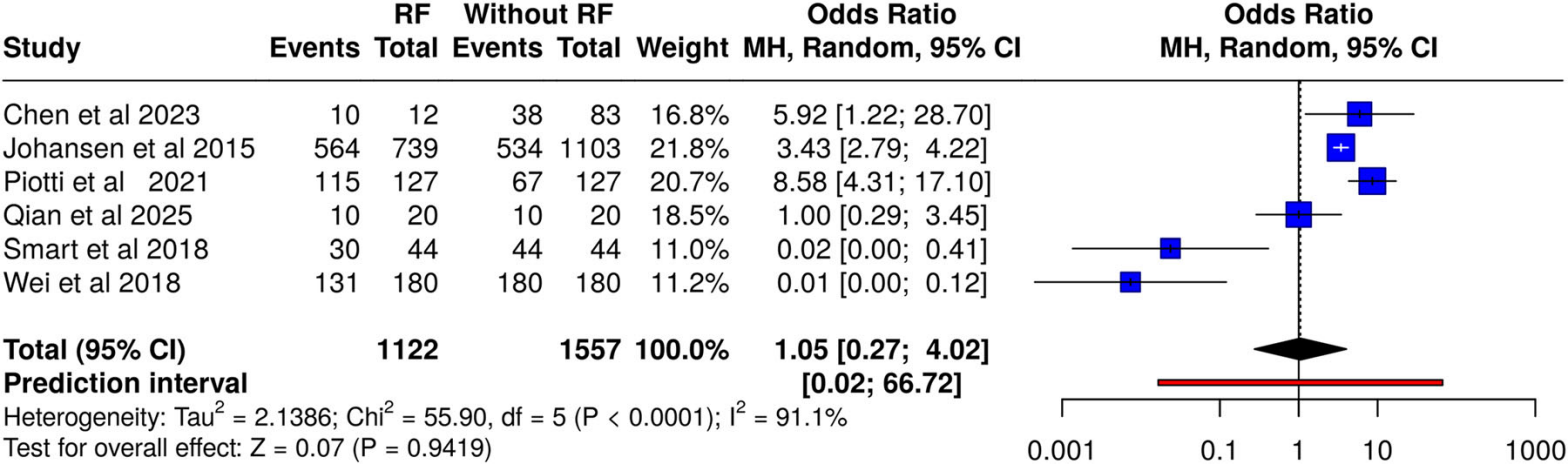
Association between elevated syndecan‐1 levels and respiratory failure in sepsis. Random‐effects model; OR = 1.05 [0.27–4.02]; *I*² = 91.1%.

Six studies (*n* = 2679 patients, including 1122 with respiratory failure and 1557 without respiratory failure) showed no significant association between syndecan‐1 and respiratory failure (OR 1.05, 95% CI, 0.27–4.02, *p* = NS) (Figure 5). Extreme heterogeneity was noted (*I*² = 91%, *p* < 0.01), indicating divergent study outcomes.

### 3.7 Endocan and Mortality in Sepsis

**Figure 6:**
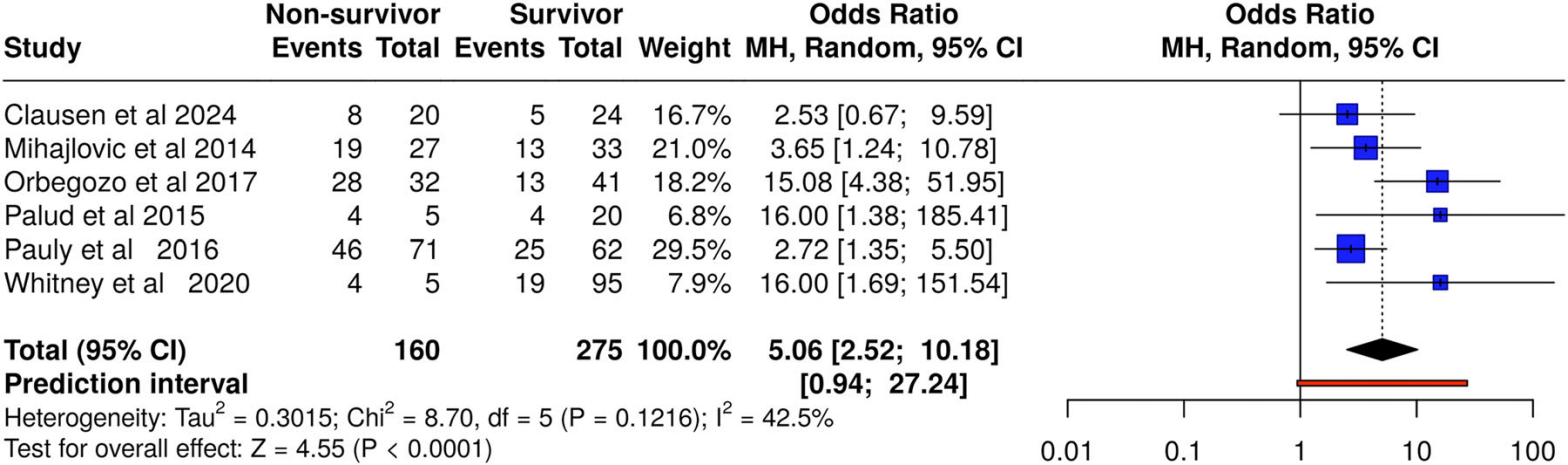
Association between elevated endocan levels and mortality in sepsis. Random‐effects model; OR = 5.06 [2.52–10.18]; I² = 42.5%.

Six studies (n = 435 patients, including 160 non‐survivors and 275 survivors) demonstrated a strong association between elevated endocan levels and mortality (OR 5.06, 95% CI, 2.52–10.18, *p* < 0.0001) (Figure 6). Unlike syndecan‐1, no significant heterogeneity was observed (*I*² = 42.5), supporting consistent effects across studies.

## 4 Discussion

This systematic review and meta‐analysis evaluated the association between glycocalyx damage biomarkers—specifically syndecan‐1, heparan sulfate, and hyaluronate—and endothelial activation markers such as endocan with clinical outcomes in patients with sepsis. The findings indicate that elevated levels of syndecan‐1 and endocan are significantly associated with an increased risk of mortality. However, syndecan‐1 levels did not show a significant relationship with multiple organ dysfunction syndrome or respiratory failure. These results suggest that glycocalyx degradation and endothelial activation may serve as valuable prognostic indicators in the clinical management of sepsis. It should be noted that median‐to‐mean conversion using the method by Wan et al. introduces estimation uncertainty, especially in skewed distributions, which may partially account for the high heterogeneity observed in pooled analyses. Variation in diagnostic criteria (Sepsis‐2 vs Sepsis‐3) across included studies may have contributed to heterogeneity in outcome associations.

A meta‐analysis of nine studies encompassing 2167 patients demonstrated a significant association between elevated syndecan‐1 levels and increased mortality (OR 2.04, 95% CI, 1.66–2.51, *p* < 0.05). This supports previous evidence that glycocalyx shedding is a hallmark of endothelial dysfunction in sepsis (Ostrowski et al. 2015; Beurskens et al. 2020). The high heterogeneity observed (*I*² = 84%) likely reflects variability in patient populations, sepsis severity, and biomarker measurement protocols. Recent studies, such as Qian et al (Qian et al. 2025)., reinforce these findings by showing that temporal changes in syndecan‐1 levels correlate with disease progression and mortality.

Endocan showed an even stronger and more consistent association with mortality (OR 5.06, 95% CI, 2.52–10.18, *p* < 0.05), accompanied by low heterogeneity across studies. As a proteoglycan secreted by activated endothelial cells, endocan reflects ongoing endothelial inflammation and injury (Pauly et al. 2016). Emerging evidence from studies like Chen et al (Chen et al. 2024). highlights endocan's prognostic value, particularly in septic cardiomyopathy, suggesting that it may be a more stable and reliable marker than syndecan‐1 in specific clinical contexts.

In contrast, syndecan‐1 did not show significant associations with multiple organ dysfunction syndrome (OR 2.35, 95% CI, 0.93–5.94) or respiratory failure (OR 1.05, 95% CI, 0.27–4.02), with both comparisons marked by substantial heterogeneity (*I*² = 94% and 91%, respectively). These findings indicate that while syndecan‐1 may serve as a marker of mortality risk, its utility in predicting organ dysfunction is limited. However, the substantial heterogeneity (*I*² > 90%) in these analyses warrants cautious interpretation, as variations in patient selection, timing of sample collection, and diagnostic criteria likely influenced pooled outcomes. This may be due to the multifactorial nature of organ failure in sepsis, where processes such as microvascular dysregulation, immune responses, and coagulation abnormalities also play key roles (Clausen et al. 2024; Piotti et al. 2021).

Mechanistically, the endothelial glycocalyx serves as a protective barrier, and its degradation in sepsis leads to increased vascular permeability, leukocyte adhesion, and the formation of microthrombi (Johansen et al. 2015). Syndecan‐1, a heparan sulfate proteoglycan, is shed upon endothelial damage, serving as a surrogate marker for glycocalyx injury. Endocan, on the other hand, is actively secreted during endothelial activation, providing insight into ongoing vascular inflammation (Smart et al. 2018). Recent research points to potential therapeutic strategies aimed at restoring or preserving the glycocalyx, such as heparin derivatives or plasma‐based resuscitation, which may improve clinical outcomes (Clausen et al. 2024; Chen et al. 2023).

Despite these promising findings, several limitations must be acknowledged. There was considerable heterogeneity in study design, including differences in sepsis definitions (Sepsis‐2 vs. Sepsis 3), timing of biomarker measurement, and patient characteristics. Pediatric data were limited, with only two studies including children, thereby reducing the generalizability of results to younger populations. Additionally, many studies lacked comprehensive adjustment for confounding factors such as comorbidities, fluid management strategies, or infection source control. While efforts were made to include gray literature, the possibility of publication bias remains, particularly given the underrepresentation of smaller studies with negative findings. Because only two studies involved pediatric patients, our conclusions primarily reflect adult populations. Additional pediatric investigations are needed to validate these associations.

These findings suggest that integrating glycocalyx‐related biomarkers into sepsis risk‐stratification models could enhance early prognostication and therapeutic targeting. Future trials might incorporate syndecan‐1 and endocan as enrichment biomarkers to identify patients most likely to benefit from endothelial‐protective therapies, such as heparanase inhibitors, plasma‐based resuscitation, or albumin supplementation.

Future research should prioritize standardized biomarker sampling protocols, ideally within 24 h of sepsis diagnosis, along with the use of validated and consistent assay platforms. Interventional studies evaluating glycocalyx‐protective therapies—including heparanase inhibitors, albumin supplementation, and endothelial‐targeted agents—are warranted to explore causal relationships. Moreover, the integration of multi‐marker panels combining syndecan‐1, endocan, and other endothelial biomarkers like angiopoietin‐2 may enhance prognostic accuracy (Wang et al. 2022). Finally, dedicated pediatric research is needed to better understand glycocalyx dynamics in children with sepsis.

## 5 Conclusion

In adult patients with sepsis, elevated syndecan‐1 and endocan levels are significantly associated with increased mortality in sepsis, affirming their role as prognostic biomarkers. However, the absence of significant associations with multiple organ dysfunction syndrome or respiratory failure suggests that glycocalyx injury alone does not fully explain the pathogenesis of organ dysfunction in sepsis. Continued research into endothelial biology and targeted therapeutic strategies remains essential for improving outcomes in this complex and life‐threatening condition.

## Author Contributions

Kamila R. Daniyarova, Zhanslu N. Sarkulova, and Amin Tamadon contributed equally to the conception and design of the study, systematic literature search, data extraction, and drafting of the manuscript. Ainur B. Tokshilykova and Botagoz M. Kalieva participated in data collection, quality assessment of included studies, and assisted in statistical analysis. Marat N. Sarkulov and Nadiar M. Mussin contributed to data interpretation, critical revision of the manuscript for important intellectual content, and provided clinical expertise in sepsis management. Ramazon Safarzoda Sharoffidin supervised the overall project, contributed to study conceptualization and methodology, resolved discrepancies during data extraction, and served as the corresponding author. All authors have read and approved the final version of the manuscript.

## Consent

The authors have nothing to report.

## Conflicts of Interest

The authors declare no conflicts of interest.

## Data Availability

The data presented in this study are available on request from the corresponding author.

## References

[mbo370155-bib-0001] Beurskens, D. M. , M. E. Bol , T. Delhaas , et al. 2020. “Decreased Endothelial Glycocalyx Thickness Is an Early Predictor of Mortality in Sepsis.” Anaesthesia and Intensive Care 48, no. 3: 221–228. 10.1177/0310057x20916471. 32486831 PMC7328096

[mbo370155-bib-0002] Chen, D. , H. Li , S. Huang , Z. Huang , Y. Sun , and L. Liu . 2024. “Heparanase Inhibitor Improves Clinical Study in Patients With Septic Cardiomyopathy.” Frontiers in Medicine 11: 1429109. 10.3389/fmed.2024.1429109. 39170046 PMC11335619

[mbo370155-bib-0003] Chen, T. T. , J. J. Lv , L. Chen , M. Li , and L. P. Liu . 2023. “Heparanase Inhibition Leads to Improvement in Patients With Acute Gastrointestinal Injuries Induced by Sepsis.” World Journal of Gastroenterology 29, no. 35: 5154–5165. 10.3748/wjg.v29.i35.5154. 37744293 PMC10514756

[mbo370155-bib-0004] Clausen, N. E. , C. S. Meyhoff , H. H. Henriksen , et al. 2024. “Plasma as Endothelial Rescue in Septic Shock: A Randomized, Phase 2a Pilot Trial.” TRANSFUSION 64, no. 9: 1653–1661. 10.1111/trf.17939. 38973502

[mbo370155-bib-0005] Holzmann, M. S. , M. S. Winkler , M. S. Strunden , et al. 2018. “Syndecan‐1 as a Biomarker for Sepsis Survival After Major Abdominal Surgery.” Biomarkers in Medicine 12, no. 2: 119–127. 10.2217/bmm-2017-0231. 29327601

[mbo370155-bib-0006] Ikeda, M. , H. Matsumoto , H. Ogura , et al. 2018. “Circulating syndecan‐1 Predicts the Development of Disseminated Intravascular Coagulation in Patients With Sepsis.” Journal of Critical Care 43: 48–53. 10.1016/j.jcrc.2017.07.049. 28843664

[mbo370155-bib-0007] Inkinen, N. , V. Pettilä , P. Lakkisto , et al. 2019. “Association of Endothelial and Glycocalyx Injury Biomarkers With Fluid Administration, Development of Acute Kidney Injury, and 90‐day Mortality: Data From the Finnaki Observational Study.” Annals of Intensive Care 9, no. 1: 103. 10.1186/s13613-019-0575-y. 31512003 PMC6738365

[mbo370155-bib-0008] Ioakeimidou, A. , E. Pagalou , M. Kontogiorgi , et al. 2017. “Increase of Circulating Endocan over Sepsis Follow‐Up Is Associated With Progression Into Organ Dysfunction.” European Journal of Clinical Microbiology & Infectious Diseases 36, no. 10: 1749–1756. 10.1007/s10096-017-2988-6. 28455780 PMC7101577

[mbo370155-bib-0009] Johansen, M. E. , P. I. Johansson , S. R. Ostrowski , et al. 2015. “Profound Endothelial Damage Predicts Impending Organ Failure and Death in Sepsis.” Seminars in Thrombosis and Hemostasis 41, no. 1: 016–025. 10.1055/s-0034-1398377. 25590523

[mbo370155-bib-0010] Lipowsky, H. H. 2012. “The Endothelial Glycocalyx as a Barrier to Leukocyte Adhesion and Its Mediation by Extracellular Proteases.” Annals of Biomedical Engineering 40, no. 4: 840–848. 10.1007/s10439-011-0427-x. 21984514 PMC3306510

[mbo370155-bib-0011] Mihajlovic, D. M. , D. F. Lendak , S. V. Brkic , et al. 2014. “Endocan Is Useful Biomarker of Survival and Severity in Sepsis.” Microvascular Research 93: 92–97. 10.1016/j.mvr.2014.04.004. 24769132

[mbo370155-bib-0012] Nelson, A. , I. Berkestedt , A. Schmidtchen , L. Ljunggren , and M. Bodelsson . 2008. “Increased Levels of Glycosaminoglycans During Septic Shock: Relation to Mortality and the Antibacterial Actions of Plasma.” Shock 30, no. 6: 623–627. 10.1097/SHK.0b013e3181777da3. 18497712

[mbo370155-bib-0013] Nelson, A. , J. Johansson , J. Tydén , and M. Bodelsson . 2017. “Circulating Syndecans During Critical Illness.” APMIS 125, no. 5: 468–475. 10.1111/apm.12662. 28256016

[mbo370155-bib-0014] Orbegozo, D. , L. Rahmania , M. Irazabal , et al. 2017. “Endocan as an Early Biomarker of Severity in Patients With Acute Respiratory Distress Syndrome.” Annals of Intensive Care 7, no. 1: 93. 10.1186/s13613-017-0311-4. 28884313 PMC5589715

[mbo370155-bib-0015] Ostrowski, S. R. , N. Haase , R. B. Müller , et al. 2015. “Association Between Biomarkers of Endothelial Injury and Hypocoagulability in Patients With Severe Sepsis: A Prospective Study.” Critical Care 19, no. 1: 191. 10.1186/s13054-015-0918-5. 25907781 PMC4423170

[mbo370155-bib-0016] Palud, A. , E. Parmentier‐Decrucq , J. Pastre , N. De Freitas Caires , P. Lassalle , and D. Mathieu . 2015. “Evaluation of Endothelial Biomarkers as Predictors of Organ Failures in Septic Shock Patients.” Cytokine 73, no. 2: 213–218. 10.1016/j.cyto.2015.02.013. 25794660

[mbo370155-bib-0017] Pauly, D. , S. Hamed , M. Behnes , et al. 2016. “Endothelial Cell‐Specific molecule‐1/endocan: Diagnostic and Prognostic Value in Patients Suffering From Severe Sepsis and Septic Shock.” Journal of Critical Care 31, no. 1: 68–75. 10.1016/j.jcrc.2015.09.019. 26489483

[mbo370155-bib-0018] Piotti, A. , D. Novelli , J. M. T. A. Meessen , et al. 2021. “Endothelial Damage in Septic Shock Patients as Evidenced by Circulating syndecan‐1, sphingosine‐1‐phosphate and Soluble VE‐Cadherin: A Substudy of ALBIOS.” Critical Care 25, no. 1: 113. 10.1186/s13054-021-03545-1. 33741039 PMC7980645

[mbo370155-bib-0019] Qian, X. , K. Y. Lui , X. Hu , et al. 2025. “Dynamic Changes and Prognosis Value of Plasma syndecan‐1 and Different Microcirculatory Parameters in Sepsis: A Prospective Observational Study.” World Journal of Surgery 49, no. 2: 353–363. 10.1002/wjs.12452. 39681545

[mbo370155-bib-0020] Reitsma, S. , D. W. Slaaf , H. Vink , M. A. M. J. van Zandvoort , and M. G. A. oude Egbrink . 2007. “The Endothelial Glycocalyx: Composition, Functions, and Visualization.” Pflügers Archiv ‐ European Journal of Physiology 454, no. 3: 345–359. 10.1007/s00424-007-0212-8. 17256154 PMC1915585

[mbo370155-bib-0021] Rudd, K. E. , S. C. Johnson , K. M. Agesa , et al. 2020. “Global, Regional, and National Sepsis Incidence and Mortality, 1990‐2017: Analysis for the Global Burden of Disease Study.” Lancet 395, no. 10219: 200–211. 10.1016/s0140-6736(19)32989-7. 31954465 PMC6970225

[mbo370155-bib-0022] Schmidt, E. P. , Y. Yang , W. J. Janssen , et al. 2012. “The Pulmonary Endothelial Glycocalyx Regulates Neutrophil Adhesion and Lung Injury During Experimental Sepsis.” Nature Medicine 18, no. 8: 1217–1223. 10.1038/nm.2843. PMC3723751 22820644

[mbo370155-bib-0023] Smart, L. , E. Bosio , S. P. J. Macdonald , et al. 2018. “Glycocalyx Biomarker syndecan‐1 Is a Stronger Predictor of Respiratory Failure in Patients With Sepsis Due to Pneumonia, Compared to Endocan.” Journal of Critical Care 47: 93–98. 10.1016/j.jcrc.2018.06.015. 29936329

[mbo370155-bib-0024] Smart, L. , S. P. J. Macdonald , S. Burrows , E. Bosio , G. Arendts , and D. M. Fatovich . 2017. “Endothelial Glycocalyx Biomarkers Increase in Patients With Infection During Emergency Department Treatment.” Journal of Critical Care 42: 304–309. 10.1016/j.jcrc.2017.07.001. 28822340

[mbo370155-bib-0025] Wang, S. , G. Liu , L. Chen , et al. 2022. “Effects of Shenfu Injection on Sublingual Microcirculation in Septic Shock Patients: A Randomized Controlled Trial.” Shock 58, no. 3: 196–203. 10.1097/shk.0000000000001975. 35959775

[mbo370155-bib-0026] Wei, S. , E. G. Rodriguez , R. Chang , J. B. Holcomb , L. S. Kao , and C. E. Wade . 2018. “Elevated Syndecan‐1 After Trauma and Risk of Sepsis: A Secondary Analysis of Patients From the Pragmatic, Randomized Optimal Platelet and Plasma Ratios (PROPPR) Trial.” Journal of the American College of Surgeons 227, no. 6: 587–595. 10.1016/j.jamcollsurg.2018.09.003. 30243993 PMC6252161

[mbo370155-bib-0027] Whitney, J. E. , B. Zhang , N. Koterba , et al. 2020. “Systemic Endothelial Activation Is Associated With Early Acute Respiratory Distress Syndrome in Children With Extrapulmonary Sepsis.” Critical Care Medicine 48, no. 3: 344–352. 10.1097/ccm.0000000000004091. 32058372 PMC8749338

